# Thinning can increase shrub diversity and decrease herb diversity by regulating light and soil environments

**DOI:** 10.3389/fpls.2022.948648

**Published:** 2022-08-05

**Authors:** Jiatong Yu, Xinna Zhang, Chengyang Xu, Minhui Hao, CholHo Choe, Huaijiang He

**Affiliations:** ^1^The Key Laboratory for Silviculture and Conservation of Ministry of Education, Key Laboratory for Silviculture and Forest Ecosystem of State Forestry and Grassland Administration, Research Center for Urban Forestry, Beijing Forestry University, Beijing, China; ^2^The Key Laboratory for Silviculture and Conservation of Ministry of Education, Beijing Forestry University, Beijing, China; ^3^Faculty of Life Science, Kim Il Sung University, Pyongyang, North Korea; ^4^Jilin Provincial Academy of Forestry Sciences Faculty of Life Science, Changchun, China

**Keywords:** mixed broadleaf-conifer forest, thinning intensity, light environment, soil nutrient content, understory species diversity

## Abstract

Tree thinning affects the light environment, which in turn affects the growth and survival of understory vegetation, thus improving species diversity and nutrient cycling, as well as the ecological habitat factors. However, the response of understory vegetation to the thinning intensity and short-time effects in the temperate broadleaf-conifer mixed forest is not completely clear. In this study, four permanent plots with a total area of 4 hm^2^ were established in a mixed broadleaf-conifer forest in northeast China, with thinning intensities of 20% (light thinning, LT), 35% (medium thinning, MT), 55% (heavy thinning, HT) and the unthinned plot (CK), respectively, in accordance with the basal area. The responses of species diversity to changes in understory vegetation were conducted by a structural equation model (SEM). The results showed that compared with CK, thinning significantly increased the photosynthetically active radiation (PAR) and the light quality (R/FR) (*p* < 0.05), while decreased the contents of soil total nitrogen (TN), total phosphorous (TP), organic matter (OM), nitrate nitrogen (NN), ammonia nitrogen (AN) and *pH*. The degree of fragmentation of light factors among the treatment plots gradually decreased as thinning intensity increased. Among all the thinning treatments, PAR and R/FR were found to be the optimal light condition when the forest thinning intensity was 55%. The light condition was found to have a significant negative correlation with soil TN, TP, OM, and AN. While the soil nutrients were positively correlated with herbaceous layer diversity but negatively correlated with shrub layer diversity. The soil nutrients were lost after thinning in a short time and herb diversity decreased, but shrub diversity increased significantly compared with unthinned plots. For the understory vegetation, the species diversity of shrub and herb layer were showed to be more sensitive to soil nutrients than light environment.

## Introduction

Plants with a height lower than 1.3m in the forest are considered understory vegetation, playing a crucial role in the species diversity of the forest ecosystem. Moreover, understory vegetation is of great significance for the nutrient circulation and energy flow of the forest ecosystem, which can change the forest microclimate (Ramovs and Roberts, 2003; Hart and Chen, 2006; Rivaie, 2014; Su et al., 2022). The dynamic adaptation of understory species diversity to the environment can affect the composition and structure of the community and reflect the development stage of the community (Ares et al., 2010). Higher species diversity is conducive to improving the stability of the forest ecosystem (Rota et al., 2014). The availability and heterogeneity of resources have long been regarded as the factors that affect the species’ coexistence, thus altering species diversity (Stein et al., 2014). However, due to the asymmetric consumption of space, light, water, and soil nutrients by the overstory, understory vegetation often exists in a limited resource environment (Su et al., 2019). Previous studies have confirmed that light and soil conditions are generally considered to be the main limiting resources affecting the establishment and growth of understory vegetation (Nicotra et al., 1999; Stein et al., 2014; Zhou et al., 2016; Su et al., 2019; Liu et al., 2021). The reasons for these inconsistent results may be related to stand types, and for the mixed broadleaf-conifer forest, the response of vegetation diversity to light and soil environments was still unclear.

Thinning has been one of the most extensively used forest management by forest managers, and it is essential management to facilitate natural forest structure (Zhu et al., 2010; Verschuyl et al., 2011). Numerous studies have shown that selective logging is capable of reducing stand density, increasing canopy opening, and leading to significant changes in the microenvironment, which has been found as the major factors for forest development (Hale, 2003; Zu-gen et al., 2005; Sercu et al., 2017). Sunlight incident to the ground surface is blocked due to the high canopy density of the unthinned stand, while the canopy structure of the thinned stand can allow a greater amount of light to reach the forest ground surface (Zhu et al., 2003; Zu-gen et al., 2005). Similarly, Nicotra et al. (1999) suggested that thinning can change the availability and spatial patterns of the below-canopy light environment. Noguchi et al. (2017) reported that forest thinning could significantly increase the light intensity in the understory, and the ratio of red to far-red light (R/FR) increased as well. Except for the light environment, thinning has also been shown to affect the physical and chemical properties of soil conditions. Lots of studies argued that soil properties are improved through thinning, e.g., the improvement of soil aeration and water permeability (the increase of soil capillary porosity and total porosity), the contents of soil organic carbon (SOC), soil total nitrogen (TN), total phosphorous (TP), nitrate nitrogen (NN) are significantly increased compared with those of the stand without thinning (Zhou et al., 2016; Dang et al., 2018). However, thinning reduced litter and nutrient return, it may reduce the content of soil nutrients (Choe et al., 2021), especially for the content of nitrogen (N) and phosphorus (P), which were primarily input through litter and reported to be the most common limiting elements in terrestrial ecosystems (Zhou et al., 2021). Therefore, the time after thinning and the thinning intensity may matter most for analyzing how thinning affects soil nutrient content.

How does understory vegetation diversity respond to changes in light and soil environment caused by thinning? One long-standing generalization in ecological research is that thinning created forest open space, improved the light environment in the forest, and increased the plant available resources, which contributed to the changes in understory vegetation diversity (Nicotra et al., 1999; Lencinas et al., 2011; Noguchi et al., 2017; Goodwin et al., 2018; Rossman et al., 2018). Another opposing view is that the improvement of light after thinning will reduce the diversity of understory vegetation. There are two main explanations: (1) In the cases of light-intensity thinning, the light environment changes a little but also produced forest gaps, which would lead to the death of shade-tolerant species (Wang et al., 2021); (2) As for the high-intensity thinning, the understory light is significantly enhanced, while one or few responsive species would quickly monopolize resources, and the other species died from the competition (Newman, 1973; Alaback and Herman, 1988; Thomas et al., 1999). Meanwhile, Soil properties are an important factor driving the distribution pattern of small-scale vegetation species (Siefert et al., 2012), and its influence on plant species diversity is complicated and does not show consistent regularity (Ning et al., 2019). Some studies believed that soil organic matter (SOM) content is a good indicator of soil fertility and nutrient availability, which tend to positively influence plant diversity (Goodall, 1980; Fu et al., 2004). However, Lenière and Houle (2006) found a weak negative relationship between organic matter (OM), TN, and herbaceous layer plant diversity. Some suggest that soil *pH* affects the activities of many enzymes involved in the mineralization of essential nutrients (N, S, and P), which is related to understory (Palmer, 1991; Chytrý et al., 2003; Weiher et al., 2004). It is well known that in the short term after thinning, only the soil surface layer is significantly affected. Besides, the root system of the herb layer is shallower than that of the shrub layer (Agnelli et al., 2004). Therefore, Rodríguez-Loinaz et al. (2008) reported that soil physicochemical properties tend to significantly positively correlate with the diversity of the herb layer, but not with shrubs. How do habitat factors among light and soil environments affect the understory species diversity? And whether the responses of the herb layer and shrub layer to these factors consistent?

In this study, three different thinning intensities were designed in the mixed broadleaf-conifer forest in northeast China. After thinning for three years, the main habitat factors (e.g., light and soil) were monitored, and the species diversity of shrubs and herbaceous were also investigated. We have discussed the effects of thinning on the soil environment and understory herbaceous diversity before (Choe et al., 2021), but the changes in light environment and shrub diversity were still unclear. Based on the “Niche Partitioning Theory” (NPT), which indicates that abiotic factors have essential roles in regulating species diversity (Austin, 1989; Pacala et al., 1996; Sterck et al., 2011), we constructed our conceptual model guided by the theory and related previous studies (Supplementary Figure 1) (Gerhardt, 1996; Lee et al., 1997; Nicotra et al., 1999; Wetzel and Burgess, 2001; Ediriweera et al., 2008). The data were analyzed using a structural equation model (SEM) to explore (1) the response of both light and soil environment in the mixed broadleaf-conifer forest to different thinning intensities, as well as (2) the influencing mechanism of light and soil environment on the species diversity of understory vegetation after thinning.

## Materials and methods

### Site description

**FIGURE 1:**
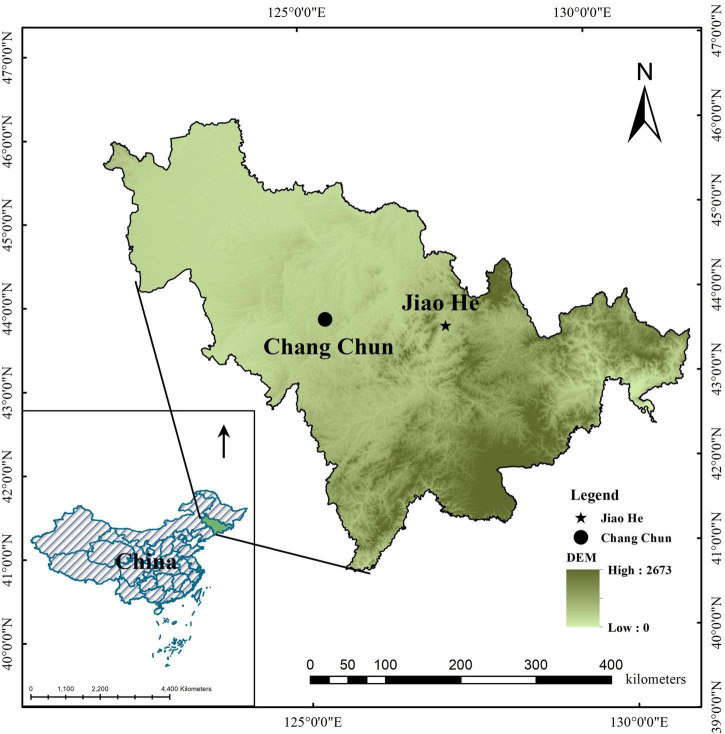
Geographical location map of the study area.

This study was carried out in the Jiaohe experimental forest (43°51′–44°05′N, 127°35′–127°51′ E, 459m a.s.l), in Jilin Province (Figure 1). The region is characterized by a typical temperate continental monsoon climate with a mean annual precipitation of 800 mm. The average annual temperature is 3.8°C, the hottest month is in July with the average temperature 23.3°C, average temperature of coldest month in January is −15°C. The soil is dark brown forest soil, and the depth range is 20–100 cm. The study site is in a mixed broad-leaved and conifer forest, dominated by *Pinus koraiensis, Tilia amurensis*, *Quercus mongolica*, *Fraxinus mandshurica*, *Carpinus cordata, Acer mono* and *Ulmus davidiana var*. *japonica*. The main shrubs are *Philadelphus schrenkii*, *Deutzia amurensis*, *Corylus mandshurica*, *Lonicera japonica* and *Eleutherococcus Senticosus*. The main herb species include *Rosaceae Filipendula*, *Aegopodium alpestre*, *Impatiens no-litangere*, *Meehania urticaefolia*, *Brachybotrys paridiformis*, *Carex pilosa Scop*, etc.

### Study site and thinning treatment

In this study, four permanent plots of 1 hectare were set, which were interfered with by thinning with different intensities (the intensity of thinning was obtained in accordance with the sectional area of chest height) in the winter of 2011. The four treatments were no thinning (CK, control), low-intensity thinning (LT, 20% of the trees removed), moderate-intensity thinning (MT, 35% of the trees removed) and high-intensity thinning (HT, 55% of the trees removed). The four plots were near-mature forests, and the information about sampling plots before and after thinning see (Table 1). To acquire more effective information and to be consistent with the principle of model parameter estimation, the grid method (Diggle and Giorgi, 2016; Choe et al., 2021) was adopted to divide the 1-hectare fixed plot (100 m × 100 m) into 400 sub-plots with equal spacing of 5 m × 5 m. The respective small quadrat within each plot was numbered as 1–400, and then 200 quadrats were randomly selected by random number method from each plot to investigate the light factors and plant species. The relative coordinate positions of the respective sampling point were recorded.

**TABLE 1:** The basic situation of plots with different thinning intensity before and after thinning.

Plots	Altitude/m	Gradient/(°)	Aspect	Before thinning				After thinning			
				Density/ (individual⋅hm^–2^)	Mean DBH/cm	Mean H/m	Base are/m^2^	Density/ (individual⋅hm^–2^)	Mean DBH/cm	Mean H/m	Base are/m^2^
CK	453	1	NE	1106	14.6	9.7	30.0652	1106	14.6	9.7	30.065
LT	443	4	NE	1045	13.9	9.6	29.4726	844	13.77	9.8	24.391
MT	430	5	NE	1007	14.8	9.7	30.3823	726	14.83	9.9	19.827
HT	447	3	NE	1298	12.4	8.8	30.4740	717	12.69	8.9	14.668

The pre-thinning survey was conducted in July 2011 and the post-thinning survey was conducted in December 2011. N, Number of trees in each plot; NE, Northeast.

### Measurement and sampling

Light factors (PAR, R/FR and NDVI), soil nutrients (TN, TP, TK, AN, NN, OM and *pH*) and understory species diversity (Shannon-Wiener Diversity index) were investigated in four plots with different thinning intensities. The restoration time of the thinning plots was 3 years, and the investigation of the four plots was from July 2014 to September 2015.

#### Light measurements

The light factors were determined using the 8-channel version of the SpectroSense2 Surface Vegetation Spectrometer (SpectroSense2) based on clear weather conditions (with cloudy or rainy weather avoided). Three optical sensor probes were used, which consisted of PAR, infrared and far-infrared, and these probes were fixed at a height of nearly 1m during the investigation. The measurement began at the same time after the quadrat was selected, and the observation position was obtained. Subsequently, the corresponding data of infrared, far-infrared, and PAR were read directly from the LED screen of the instrument.

The measured data could be exported by software. Moreover, the data could be edited and counted in Excel and Word. The average value of PAR, R/FR (red/far-red) was used for calculation (light quality). Next, the average value was obtained. Normalized vegetation index (NDVI) refers to a transformation form of near-infrared and RED channel reflectance ratio (SR = NIR/RED), NDVI = (NIR-R)/(NIR+R).

#### Soil sampling

We divided each 1-hectare plot (100 m × 100 m) into 4 sub-plots with equal spacing of 50 m × 50 m for soil samples, and 25 sampling sites were sited in each sub-plots. The samples of 0–20 cm soil layer were collected using a ring knife. Plant residues, gravel and other sundries in the unearthed samples were selected, and the fresh soil samples were dried and screened. The *pH* was obtained using the electrode potential method (Richards, 1954). Organic matter (OM) was obtained using the potassium dichromate volumetric method in combination with the external heating method (Walkley and Black, 1934). Total nitrogen (TN) was determined by the Kjeldahl method. Total phosphorus (TP) was determined by the Mo-Sb colorimetric method (Li and Wang, 2012). Total potassium (TK) was obtained through NaOH melting flame photometry. Soil ammonium nitrogen (AN) and nitrate nitrogen (NN) were extracted with potassium chloride and then measured with the flow analyzer (FIA) (Wilke et al., 2005).

#### Shrub and herb diversity

The understory species diversity in plots was investigated using the ShannonWiener Diversity index (H). The species diversity is expressed as:


$$H=-\Sigma \,P_{i}\,l\,n\,P_{i}$$

$$P_{i}=\frac{N_{i}}{N}$$

*N*_*i*_ denotes the number of the *i-*th species; *N* represents the number of all intermediate individuals.

### Statistical analyses

A Generalized Linear Mixed Model (GLMM) and One-way ANOVA to analyze the effect of thinning intensities on light condition, soil nutrients and understory species diversity. The Kolmogorov-Smirnov (K-S) test was performed to examine the normality of the data. The data not consistent with the normal distribution was converted. The habitat factors (PAR, R/FR, NDVI, TN, TP, TK, *pH*, OM, AN, NN) were extracted using the PCA into a few principal components and represented the light and soil environments in subsequent analyses first (García-Palacios et al., 2017; Ruiz-Benito et al., 2017; Hao et al., 2020). And a SEM (Supplementary Figure 1) were developed to explore how habitat factors (light and soil environment) affect the shrub and herb diversity. Besides, Bentler’s comparative fit index (CFI) and root mean square error of approximation (RMSEA) were employed as the fitting goodness indicator. The principal component analysis and the SEM were conducted with software SPSS26.0 and Amos, respectively. All statistical analyses were performed using the R software. The GLMM was implemented using the “lme4” package (Lingjun et al., 2019), and SEM using the “lavaan” package (Rosseel, 2012). Kriging interpolation mapping was generated using the software of ArcGIS10.2.

## Results

### Effects of thinning on light factors

#### Effects of different thinning intensities on light factors

**FIGURE 2:**
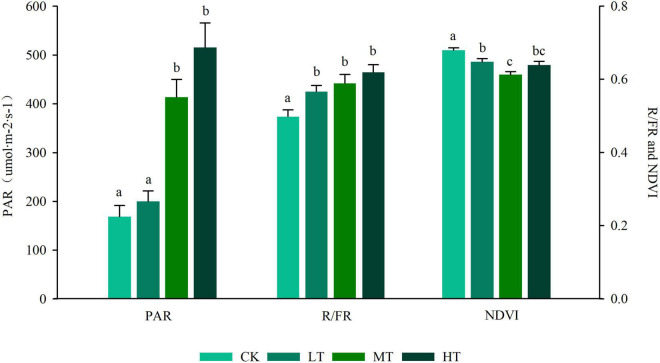
Response of light factors on the different strength of thinning. The left ordinate represents photosynthetically active radiation (PAR), and the right represents normalized vegetation index (NDVI) and R/FR. Different letters of the mean values represent significant differences (*p* < 0.05). CK, unthinned plot; LT, thinning intensity was 20%; MT, thinning intensity was 35%; HT, thinning intensity was 55%.

The GLMM and ANOVA results showed that there were significant differences among the light factors in different thinning intensities (Figure 2 and Supplementary Table 1). The mean value of PAR ranged from 169.0 (CK) to 515.46 (HT), and the mean value increased with the increase in the thinning intensity, whereas no significant difference was found between the control group and the low-intensity thinning group (LT). Moreover, the control group and the low-intensity thinning group were significantly lower than the moderate and high thinning groups (MT and HT), and no significant difference was found in PAR between the moderate and high thinning groups. The mean value of R/FR ranged from 0.499 (CK) to 0.628 (HT), and it increased monotonically as the thinning intensity increased. The mean value of R/FR in the control group was significantly lower than that in the thinning group, whereas no significant difference was identified in the mean value of R/FR between the three thinning groups. The lowest Normalized vegetation index (NDVI) was 0.613 ± 0.0083 when the thinning intensity was 35%.

#### Spatial distribution pattern of light factors

**FIGURE 3:**
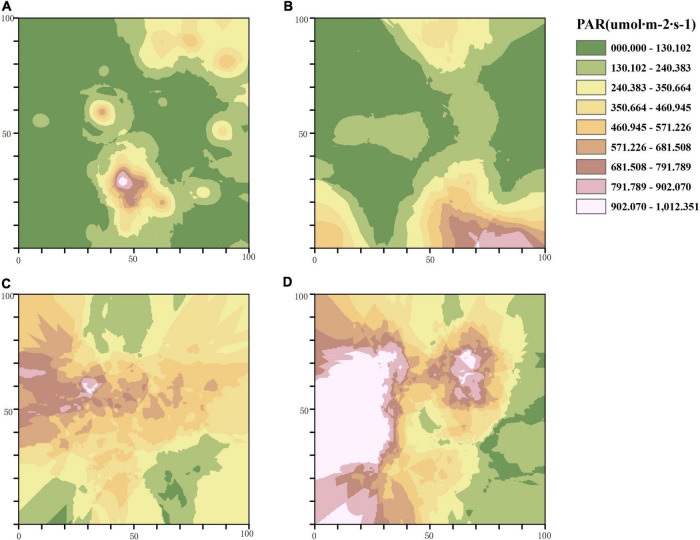
Spatial distribution pattern of photosynthetically active radiation (PAR). **(A)** Unthinned plot (CK), **(B)** light thinning plot (LT), **(C)** medium thinning plot (MT), **(D)** heavy thinning plot (HT).

**FIGURE 4:**
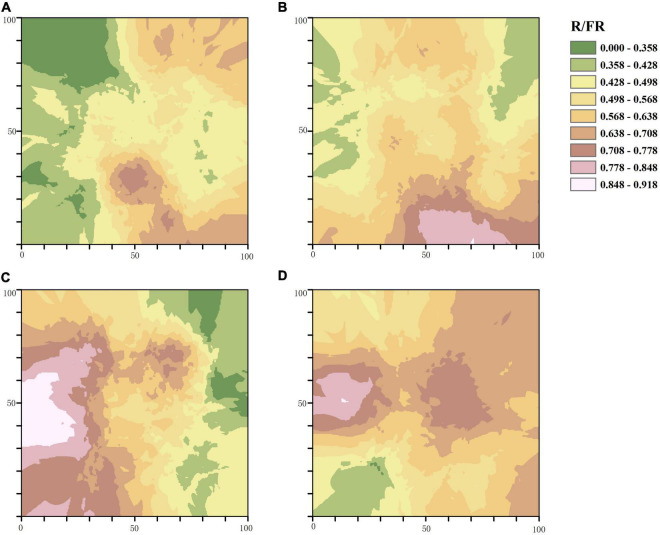
Spatial distribution pattern of R/FR. **(A)** Unthinned plot (CK), **(B)** light thinning plot (LT), **(C)** medium thinning plot (MT), **(D)** heavy thinning plot (HT).

In accordance with the semi-variogram theory and the structural analysis, the kriging method was used to optimize the interpolation of the sampling area and generate the isoline map of light factors in the stand with different thinning intensities. The distribution of PAR in the control group was patchy with a high fragmentation degree, and most areas of the stand received less PAR. With the increase of thinning intensity, the area that can receive light in the treatment stands gradually expands, and the degree of PAR fragmentation gradually decreases. The area of PAR higher than 460.945μmol⋅m^–2^⋅s^–1^ accounted for two thirds of the total area of the sample plot characterized by high-intensity thinning (Figure 3). The distribution of R/FR in different thinning intensities was patchy. The degree of R/FR fragmentation in the CK and the MT plots was higher than that of the LT and HT plots, while the area with high R/FR (greater than 0.848) in the MT plot was the largest among the four plots (Figure 4).

### Effects of thinning on soil conditions

Different thinning intensities exerted different effects on soil nutrient indexes (Table 2 and Supplementary Table 1). The TN content in soil tended to decrease with the increase of the cutting intensity. Besides, a significant difference was found between the CK group and thinning treatments, whereas there was no significant difference among the three treatment groups. Significant differences were found in TP and NN between the CK and thinning treatments. The F values of *pH*, OM and AN contents were 30.598, 31.856 and 25.017, respectively. Furthermore, Sig values were all lower than 0.01, thus suggesting that different thinning intensities significantly affected *pH*, OM and AN. The F value and Sig value of TK were 2.032 and 0.109, thus revealing that different thinning intensities did not significantly affect TK (*p* < 0.05).

**TABLE 2:** One-factor ANOVA of soil nutrient.

Factors	CK	LT	MT	HT
TN g/Kg	8.78(0.37)a	5.08(0.31)b	4.79(0.18)b	4.11(0.30)b
TP g/Kg	0.90(0.03)a	0.53(0.05)b	0.56(0.02)b	0.54(0.03)b
TK g/Kg	8.77(0.18)a	8.41(0.19)a	8.44(0.16)a	8.20(0.15)a
pH	5.53(0.02)a	5.25(0.02)b	5.38(0.03)c	5.27(0.02)b
OM g/Kg	12.49(0.42)a	9.07(0.50)b	7.90(0.40)bc	7.24(0.37)c
AN mg/Kg	3.89(0.13)a	4.40(0.18)a	3.09(0.10)b	3.07(0.09)b
NN mg/Kg	18.40(0.75)a	6.30(0.46)b	6.74(0.37)b	6.87(0.50)b

Data are mean values (standard error). CK: unthinned plot, LT: light thinning plot, MT: medium thinning plot, HT: heavy thinning plot. Different lowercase letters indicate significant differences among plots (*p* < 0.05).

### Effect of thinning intensities on understory species diversity

Different thinning intensities exerted different effects on shrub and herb layers diversity (Table 3 and Supplementary Table 2). The Shannon-Wiener diversity index of herb layers in the CK group is highest, followed by the MT plot. As for the shrub layers, the CK group was the lowest compared with the thinned group (*p* < 0.05).

**TABLE 3:** One-way ANOVA of Shannon-Wiener Diversity index of understory.

Plots	Shannon-Wiener Diversity index (H)	
	Herb layers	Shrub layers
CK	1.44(0.22)a	0.11(0.36)b
LT	1.11(0.27)c	0.31(0.30)a
MT	1.23(0.28)b	0.35(0.37)a
HT	1.14(0.32)cd	0.41(0.37)a

Data are mean values (standard error). Different letters of the mean values indicate significant differences (*p* < 0.05).

### Effect of light and soil environment on understory species diversity

**FIGURE 5:**
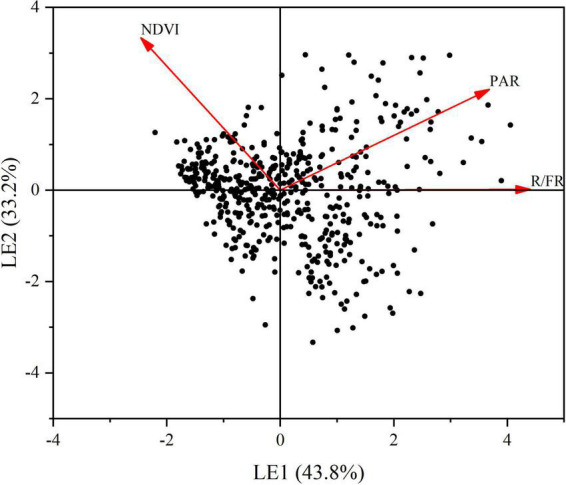
Principal component analysis (PCA) of light environment. Red arrows depict the loads of pearson correlations between light environmental variables including photosynthetically active radiation (PAR), R.FR and normalized vegetation index (NDVI). Variance explained by each principal component is shown in brackets. LE: light environment. LE1: the first axes of a principal component. LE2: the second axes of a principal component.

**FIGURE 6:**
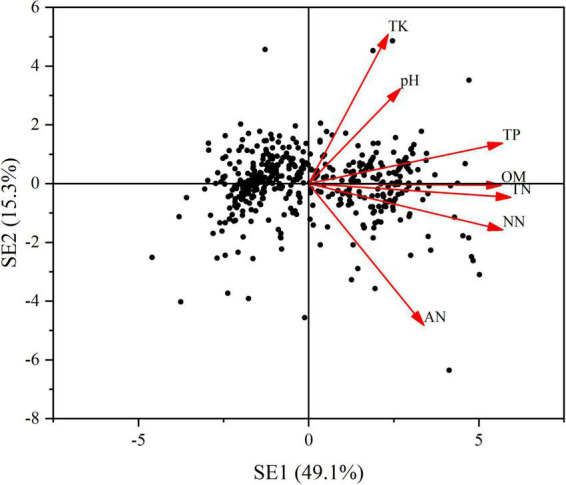
Principal component analysis (PCA) of soil nutrients. Red arrows depict the loads of pearson correlations between light environmental variables including TN, TP, TK, *pH*, OM, AN, and NN. Variance explained by each principal component is shown in brackets. SE: soil nutrients. SE1: the first axes of a principal component. SE2: the second axes of a principal component.

A principal component analysis (PCA) was conducted to compress the variation of light factors into several principal components. The first principal component (LE), which represented 43.8% of the total variation (Figure 5) was selected to represent the main information of the lighting environment. Other components with the eigenvalue less than 1 was abandoned in this analysis. Likewise, using the same procedure, we conducted a PCA to extract the main information from the seven soil properties. The first two principal components (SE1 and SE2), which jointly captured 64.4% of the total variation were retained (Figure 6). Through the principal component analysis (PCA), the dimension reduction analysis was conducted on the original habitat data. LE, SE1 and SE2 basically represented the information of light and soli environmental factors, respectively.

**FIGURE 7:**
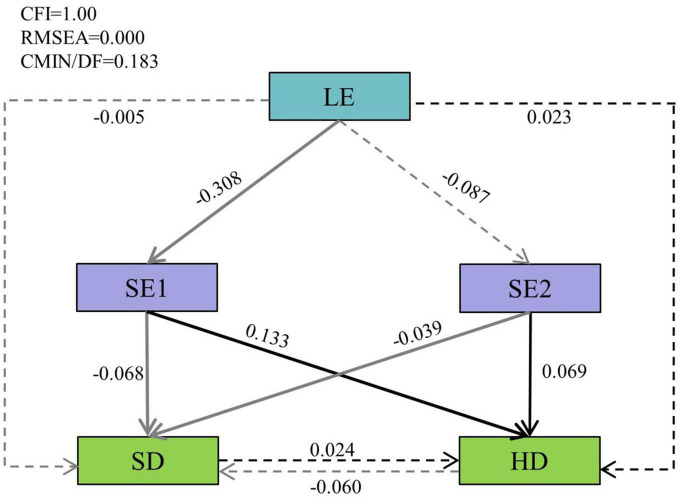
The structural equation model (SEM) results. The single-directional arrows represent the hypothesized cause-and-effect relationships between the variables. The standardized path coefficients are marked on the arrow lines. Residual errors related to the respective endogenous variable in the model are not presented. The solid lines represent significant relationships (*p* < 0.05), and dashed lines represent insignificant relationships (*p* > 0.05). Black arrows represent positive effects, and the gray arrows represent negative effects. Light factor (LE) represents the light factors extracted by the principal component analysis (PCA), the first axes of a principal component (SE1) and the second axes of a principal component (SE2) represent the first two soil factors extracted by the PCA, SD, and HD represent Shannon-Weiner index of shrub and herb, respectively.

Thus, we used these principal components to build a SEM. The results confirmed that our structural equation model had a good fit to the data (CFI = 1.00, RMSEA = 0.00, and CMIN/DF = 0.183) (Figure 7). The light factor (LE) in the stand after thinning had a significant negative effect on SE1 (the standardized path coefficients: *r* = −0.308, *p* < 0.05), while LE did not significantly affect SE2 (*r* = −0.087, *p* > 0.05). The correlation between light and Shannon-Weiner index of understory were not significant. However, the soil nutrients (SE1 and SE2) showed a negative correlation with Shannon-Weiner index of shrub but a positive correlation with Shannon-Weiner index of herb. The relationship between shrubs and herbs was not significant. Furthermore, light indirect affects shrub and herb Shannon-Weiner index by directly changing soil conditions.

## Discussion

### Effects of thinning on habitat factors

Thinning is a vital measure to adjust stand structure. The change in canopy structure that arises from forest thinning is the main driving mechanism of the change in the understory light environment (Richards and Hart, 2011), which mainly alters the light flux density and spectral quality, and spatial distribution through the absorption, reflection and transmission of light by the upper leaves (Parker et al., 2002). Consistent with a large number of previous studies (Montgomery and Chazdon, 2001; Wetzel and Burgess, 2001; Sullivan et al., 2002), we found that the PAR and R/FR increased monotonically with the thinning intensity. Thinning shortens the canopy structure, expands the canopy space, and the light characteristics are sensitive to the change of forest layers (Wan and He, 2020), which will reduce the obstruction of incident light and increase the par under the forest PAR (Yamada et al., 2014; Franca et al., 2018). The NDVI is the normalized vegetation index, capable of fully indicating the vegetation coverage in the forest community (Liang et al., 2018), and we found that the mean value of NDVI in HT group was higher than in the other groups. Thus, the probable reason for the above result is that the sample plots with high thinning intensities have sufficient sunshine and more weeds, thus resulting in a large vegetation coverage area and a higher NDVI value. Our results indicated that the light factors in the CK group showed significant patch distribution in space, leading to the discontinuity of light factors. The thinning forests would have more uniform light than the original forest due to its more uniform canopy structure. Similarly, Nicotra et al. (1999) and Yamada et al. (2014) reported that selectively logged stands are more homogeneous with regard to variation in light than the original forest.

As for the soil environment, most studies have suggested that soil nutrient content decreases in a short term after thinning (Jennings et al., 2012; Choe et al., 2021). Binglin et al. (2020) argued that five years after the thinning, the soil TP and TK contents of the Birch plantation decreased significantly. Thus, an increase in the logging intensity could trigger the loss of soil nutrients (N, P, and K). This study was consistent with the above research, thinning significantly reduced the TN, TP, OM, NN, and AN in the soil, whereas the change of TK content in the soil was insignificant. The reasons for the above result were speculated as follows: (1) After thinning, the amount of nutrient return through the decomposition of litters decreases, so the soil nutrient content formed by the decomposition and the transformation of litters declines (Zhou et al., 2019). (2) The removal of residues from thinning can lead to the loss of nutrients (e.g., N, P, and K) from soil (Dong et al., 2021). Thinning produces a considerable amount of nutrient-rich residues that are broken down by organisms in the soil and can deliver nutrients to the soil (Garcia-Oliva et al., 2003; Semenov et al., 2012; Wang et al., 2012). Existing studies have suggested that soil total carbon and TN increased during residue retention in 12-24 months after thinning (Chen and Xu, 2005; Tutua et al., 2019). In contrast, removing the whole plant after thinning would lead to the loss of soil carbon and nitrogen and the decrease of unstable soil OM (Kumaraswamy et al., 2014). Accordingly, compared with the removal of forest residues, the retention of forest residues can improve the quality of resources and make up for the loss of soil nutrients caused by thinning, thus facilitating plant growth and maintaining the balance of forest nutrients (Fernandez et al., 2016; Rocha et al., 2018). (3) After thinning, the change in soil temperature and water content in the stand will facilitate the decomposition and consumption of soil OM, thus resulting in the decrease of soil nutrient content (Jandl et al., 2007; James and Harrison, 2016). (4) The investigation of this study was conducted 3 years after the thinning, and the forest recovery time was relatively short. As a result, the residual effect of forest interference by thinning may still exist. Thus, long-term observation should be conducted on the effects of thinning on soil nutrients (Choe et al., 2021).

### Effect of light and soil environment on understory species diversity

The change of light conditions in the forest directly affects the physical and chemical properties of soil. Our results showed that there was a negative correlation between light and soil nutrients. Lu et al. (2020) have also drawn a similar conclusion. Light caused the formation of a large number of free radicals such as hydroxyl, peroxy, singlet oxygen and peroxide in the soil, which can accelerate the photolysis of organic matter (Mathew and Khan, 1996). And photochemical loss of organic matter can partly explain the 20–50% reduction in soil organic matter content in tillage layer, and these losses are concentrated at the top 10 cm of soil (Tiessen et al., 1982; Luo et al., 2010).

The amount of light reaching the understory is one of the most important limiting factors affecting the species of understory plants (Kirby, 1988; Van Calster et al., 2008; Márialigeti et al., 2016; Shi et al., 2021), which determines the coverage, diversity and species composition of undergrowth vegetation. Many studies have shown that improved understory light conditions caused by increased canopy openness can improve plant diversity, especially in the herbaceous layer (Lee et al., 1997; Beaudet and Messier, 2002; Vesala et al., 2005; Ligot et al., 2014; Wang et al., 2017; Ádám et al., 2018; Wan and He, 2020; Shi et al., 2021; Zhang et al., 2021; Su et al., 2022). Theoretically, the effects of light on vegetation are complex because the abundance or diversity of understory vegetation does not necessarily correlate positively with light availability. For example, in the study by Härdtle et al. (2003), light conditions were the most important driver of acid beech forests, whereas in more neutral forest sites, soil properties proved to be more decisive. In our research, we did not find significant light condition effects, and soil properties were turned out to contribute more to the diversity of undergrowth vegetation than light conditions. One supposed reason was that there were enough light resources in the understory after thinning, and the soil nutrient content became the main limiting factor for the survival of undergrowth plants.

According to the SEM obtained in this study, both SE1 and SE2 were negatively correlated with shrub layer diversity, while positively with herb layer, which means that the decrease of soil nutrients would lead to a decline in the shrub diversity, but not in herbs. Vegetation and soil are inseparable, and soil nutrients are important factors driving the species diversity of understory vegetation (Pohl et al., 2009). Previous studies had proved that understory vegetation diversity was mainly affected by soil factors, including soil *pH* (Tabatabai and Bremner, 1970; Acosta-Martínez and Tabatabai, 2000; Pärtel et al., 2004; Lenière and Houle, 2006), soil P contents (Sitters et al., 2013; Feng et al., 2018) and soil K contents, etc. (Potts et al., 2002; Bobbink et al., 2010; Santana et al., 2010), and there were different effects on the diversity of shrub and herb layers. In general, the growth of understory vegetation is related to the availability of essential soil nutrients, the solubility of toxic elements, and soil microbial communities (Weaver and Hamill, 1985). For the shrub layer, higher *pH* and soil water content can reduce the availability of soil nutrient elements (such as available phosphorus) and increase the dissolution of toxic elements, thus inhibiting the growth of soil microorganisms and some shrub plants that are alkali-resistant and sensitive to toxic elements (Ma et al., 2012). However, the herbaceous calcareous plants that prefer humidity habitats often have a tolerance to higher soil *pH* (Yibo et al., 2019). As for the soil P content, studies on plant niche differentiation along the gradient of soil nutrient resources found that niche differentiation only occurred when the P content was low, which was beneficial to the coexistence of more species (Steidinger, 2015; Xu et al., 2017). In addition, excessive soil K contents reduced the fine root biomass at the community level (Wright et al., 2011; Xu et al., 2017), which also caused damage to shrubs. All the above studies have obtained consistent results, that is, soil factors are positively correlated with herbs and negatively or not correlated with shrubs, which is consistent with our findings. Compared with a light environment, soil conditions showed a larger influence on the species diversity of understory vegetation in mixed broadleaf-conifer forest three years after thinning.

## Conclusion

The results of this study suggested that forest thinning affected the light factors and the soil conditions in the stand, and changes in the above habitat factors would have further effects on the species diversity of understory vegetation. In the short period (3 years) after thinning, in the Broad-leaved Korean pine forest of northeast China, the light environment was found as the optimal light environment in the heavy thinning forest. However, TN, TK, OM, and NN decreased with the increase of the cutting intensity. After thinning, the light factor showed a significant negative correlation with the soil environment in the stand. The response of understory shrub and herbaceous diversity to the soil environment was more significant than that of the light condition. Lower soil nutrients were related to a higher shrub diversity but a lower herb diversity. This study was conducted 3 years after the thinning, and post-felling recovery is a long-term process with various habitat factors in the stand changing constantly, thus leading to the change of understory vegetation diversity. Accordingly, it is suggested that long-term dynamic monitoring should be conducted to lay a more solid theoretical basis for forest management.

## Data availability statement

The original contributions presented in this study are included in the article/Supplementary material, further inquiries can be directed to the corresponding author.

## Author contributions

XZ, MH, and CX: conceptualization. JY: methodology and writing—original draft preparation. XZ and JY: software. XZ, CX, and CC: validation. CC and HH: investigation. XZ: writing—review and editing, project administration, and funding acquisition. All authors have read and agreed to the published version of the manuscript.
