# Supplementary material for: Thinning can increase shrub diversity and decrease herb diversity by regulating light and soil environments
Source: Front Plant Sci. 2022 Aug 5;13:948648. doi: 10.3389/fpls.2022.948648 (PMC9389291; doi:10.3389/fpls.2022.948648)
Supplement: Supplementary file 1 [file Table_1.docx]

Supplementary Material

**Supplementary Table 1.** The results of Linear Mixed Model of light and soil factors.

| **Factors** | **Plots** | **Intercept value** | **t value** |
| --- | --- | --- | --- |
| **PAR** | CK | 167.745 | 3.794* |
|  | LT | -1.026 | -0.016^ns^ |
|  | MT | 243.286 | 3.897* |
|  | HT | 366.512 | 5.870* |
| **R/FR** | CK | 0.498 | 19.879* |
|  | LT | -0.001 | -0.028^ns^ |
|  | MT | 0.103 | 2.918* |
|  | HT | 0.124 | 3.512* |
| **NDVI** | CK | 0.684 | 63.079* |
|  | LT | -0.004 | -0.261^ns^ |
|  | MT | -0.070 | -4.616* |
|  | HT | -0.38 | -2.457* |
| **TN g/Kg** | CK | 8.675 | 37.112* |
|  | LT | -3.514 | -8.548* |
|  | MT | -3.682 | -11.153* |
|  | HT | -4.655 | -14.100* |
| **TP g/Kg** | CK | 0.898 | 32.241* |
|  | LT | -0.340 | -7.079* |
|  | MT | -0.324 | -8.234* |
|  | HT | -0.356 | -9.062* |
| **TK g/Kg** | CK | 8.828 | 81.541* |
|  | LT | -0.411 | -2.037* |
|  | MT | -0.467 | -3.055* |
|  | HT | -0.591 | -3.865* |
| **pH** | CK | 5.526 | 258.189* |
|  | LT | -0.278 | -8.479* |
|  | MT | -0.150 | -4.928* |
|  | HT | -0.261 | -8.652* |
| **OM g/Kg** | CK | 12.656 | 55.246* |
|  | LT | -3.514 | -8.812* |
|  | MT | -4.332 | -13.388* |
|  | HT | -5.728 | -17.700* |
| **AN mg/Kg** | CK | 3.794 | 48.754* |
|  | LT | 0.523 | 3.640* |
|  | MT | -0.733 | -6.669* |
|  | HT | -0.689 | -6.268* |
| **NN mg/Kg** | CK | 18.731 | 46.23* |
|  | LT | -12.524 | -17.16* |
|  | MT | -11.597 | -20.27* |
|  | HT | -11.717 | -20.45* |

Note: ^ns^ means not significant compared with the control group (unthinned group), t<|2|; ^*^ means significant, t>|2|

**Supplementary Table 2.** The results of Linear Mixed Model of Shannon-Wiener Diversity index of understory.

| **Shannon-Weiner index (H)** | **Plots** | **Intercept value** | **t value** |
| --- | --- | --- | --- |
| **Herb layer** | CK | 1.443 | 49.675* |
|  | LT | -0.327 | -8.085* |
|  | MT | -0.213 | -5.215* |
|  | HT | -0.303 | -6.833* |
| **Shrub layer** | CK | 2.601 | 1.564* |
|  | LT | 11.345 | 4.823* |
|  | MT | 7.324 | 3.119* |
|  | HT | 8.227 | 3.503* |

Note: ^ns^ means not significant compared with the control group (unthinned group), t<|2|; ^*^ means significant, t>|2|

**Supplementary Figure:**

**
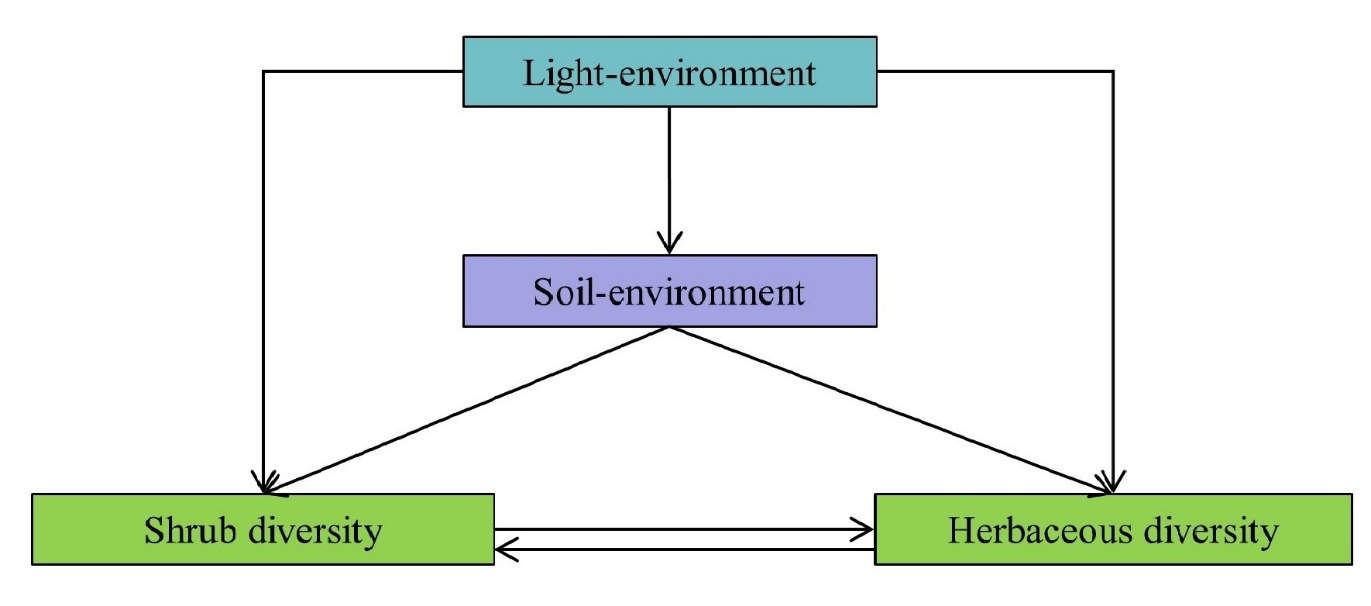
**

**Supplementary Figure 1.** A priori model. The single-directional arrows represent the hypothesized cause-and-effect relationships between the variables.
