# Supplementary figures and images for: Thinning can increase shrub diversity and decrease herb diversity by regulating light and soil environments
Source: Front Plant Sci. 2022 Aug 5;13:948648. doi: 10.3389/fpls.2022.948648 (PMC9389291; doi:10.3389/fpls.2022.948648)

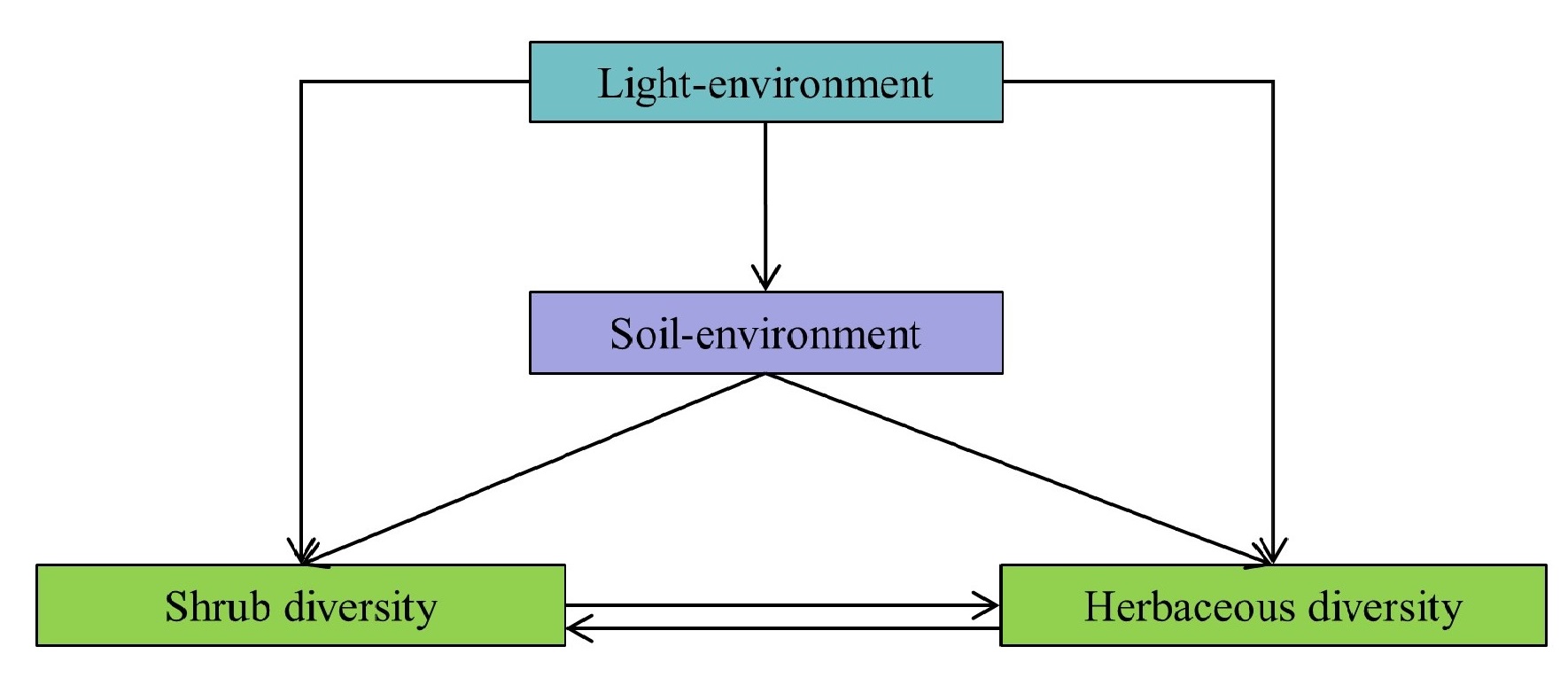

Supplement: Supplementary file 2 [file Image_1.jpg]
